# Colonic Endoscopic Tubing Is Safe and Effective Approach for Washed Microbiota Transplantation in Autistic Children

**DOI:** 10.1155/2023/7838601

**Published:** 2023-11-22

**Authors:** Qing-Fen Yuan, Hui-Yi Wu, Xian-Yun Chen, Ya-Mei Zheng, Song-Lin Fu, Xing-He Wang, Jian-Wei Zhu, Jian-Dong Guo, Xing-Xiang He, Li-Hao Wu

**Affiliations:** ^1^Department of Gastroenterology, The First Affiliated Hospital of Guangdong Pharmaceutical University, Guangzhou, Guangdong Province, China; ^2^Research Center for Engineering Techniques of Microbiota-Targeted Therapies of Guangdong Province, The First Affiliated Hospital of Guangdong Pharmaceutical University, Guangzhou, Guangdong Province, China; ^3^Inner Mongolia Ewenki Autonomous Banner People's Hospital, The Nei Monggol Autonomous Region, China

## Abstract

**Background:**

Washed microbiota transplantation (WMT) as the improved methods of fecal microbiota transplantation has been employed as a therapeutic approach for ameliorating symptoms associated with autism spectrum disorder (ASD). In this context, colonic transendoscopic enteral tubing (TET) has been utilized as a novel procedure for administering WMT.

**Methods:**

Data of children with ASD who received WMT by TET were retrospectively reviewed, including bowel preparation methods, TET operation time, success rate, tube retention time, the comfort of children, adverse events, and parent satisfaction.

**Results:**

A total of 38 participants underwent 124 colonic TET catheterization procedures. The average time of TET operation was 15 minutes, and the success rate was 100% (124/124). There was no significant difference in TET operation time between high-seniority physicians and low-seniority physicians. In 123 procedures (99%), the TET tube allowed the completion of WMT treatment for 6 consecutive days. In 118 procedures (95.2%), the tube was detached spontaneously after the end of the treatment course, and the average TET tube retention time was 8 days. There was no incidence of tube blockage during the treatment course. No severe adverse events occurred during follow-up. Parents of all participants reported a high level of satisfaction with TET.

**Conclusion:**

Colonic TET is a safe and feasible method for WMT in children with ASD.

## 1. Introduction

Autism spectrum disorder (ASD) is a heterogenous developmental disorder characterized by different levels of social disorders, speech stunting, and specific and narrow interests [1, 2]. The symptoms of ASD typically manifest at the age of three years [3]. The estimated global prevalence of ASD in 2016 was approximately 1%. There are no proven treatments for ASD [4]. Some of the relatively effective treatments for ASD include behavioral therapy, speech and social therapy, and dietary nutritional/medical treatments [5–7]; however, there is a paucity of medical treatments to treat the core symptoms of ASD [8].

Recent years have witnessed a remarkable interest in the therapeutic use of fecal microbiota transplantation (FMT) [9]. Studies have explored the use of FMT for the treatment of a variety of illnesses in children, including inflammatory bowel disease (IBD) [10], allergic colitis, and gut-brain axis diseases [11], such as autism and epilepsy [12], excluding recurrent Clostridium difficile infection (CDI) [13]. FMT has shown promising results in pediatric patients, although the available evidence is largely limited to case series and individual case reports [14]. In recent years, studies have explored the relationship between the gut microbiota and autism which is potentially mediated via the gut-brain axis [15]. Modulation of the gut microbiota through use of antibiotics, probiotics, or prebiotics and/or FMT may be a viable therapeutic option for autism [16]. For example, a trial investigated the long-term efficacy of FMT for the treatment of patients with autism (this trial used a combination of antibiotics, gut cleansing, gastric acid suppression, and FMT), and the participants were followed for two years after the completion of treatment; most of the improvements in gastrointestinal symptoms were maintained, and there was a substantial improvement in autism-related symptoms [17]. In another study, FMT in children diagnosed with ASD resulted in significant, lasting improvements in gastrointestinal symptoms and autistic behaviors by restoring normal microbial balance. These findings indicated the influence of the microbiome on gastrointestinal symptoms and autistic behaviors in children with ASD [18]. In our recent study, washed microbiota transplantation (WMT) was found to improve ASD symptoms, and we observed a positive correlation between the WMT course and the degree of improvement [19]. WMT is an automated preparation method based on an automated decontamination system (GenFMTer; FMT Medical, Nanjing, China) to enrich the gut microbiota using fecal material, which can distinctly reduce FMT-related adverse events. It has been applied in many FMT centers in China. FMT can be performed in the upper or lower gastrointestinal tract [20], and the most common administration techniques include nasogastric/nasojejunal tubes [21], oral capsules [22], esophagogastroduodenoscopy, colonoscopy, enemas, endoscopic bowel tubes, and percutaneous endoscopic cecostomy. Each of these modes of administration has their advantages and limitations. The safety, efficacy, and cost of FMT depend not only on the fecal bacteria but also on the route of administration. Transendoscopic enteral tubing (TET) is a new method of FMT proposed by Professor Faming Zhang in 2016 [17]. In 2019, Allegretti et al. of the Harvard Medical School found colonic TET to be a promising new approach for FMT [23]. However, there is no standardized approach for the selection of FMT methods. The most optimal method for clinical application in a particular setting needs to be assessed. Currently, there is a paucity of data on whether WMT by colonic TET is feasible in children with ASD. Therefore, in this study, we evaluated the safety and feasibility of colonic TET in children with ASD.

## 2. Materials and Methods

### 2.1. Participants

We retrospectively enrolled 38 children with ASD who underwent colonic TET intubation for WMT at the First Affiliated Hospital of Guangdong Pharmaceutical University between June 2019 and December 2020. The inclusion criteria were as follows: age range, 2–18 years; either sex; parental agreement for follow-up; and ASD diagnosed by the pediatrics or psychology department. Patients were excluded if they had other diseases, such as infant dementia or childhood depression. This study was conducted according to the principles of the Declaration of Helsinki and approved by the Ethics Committee of the First Affiliated Hospital of Guangdong Pharmaceutical University, Guangzhou, China (BASEC-ID 2020-06)).

### 2.2. Standard Procedure for Colonic TET Placement

**Figure 1:**
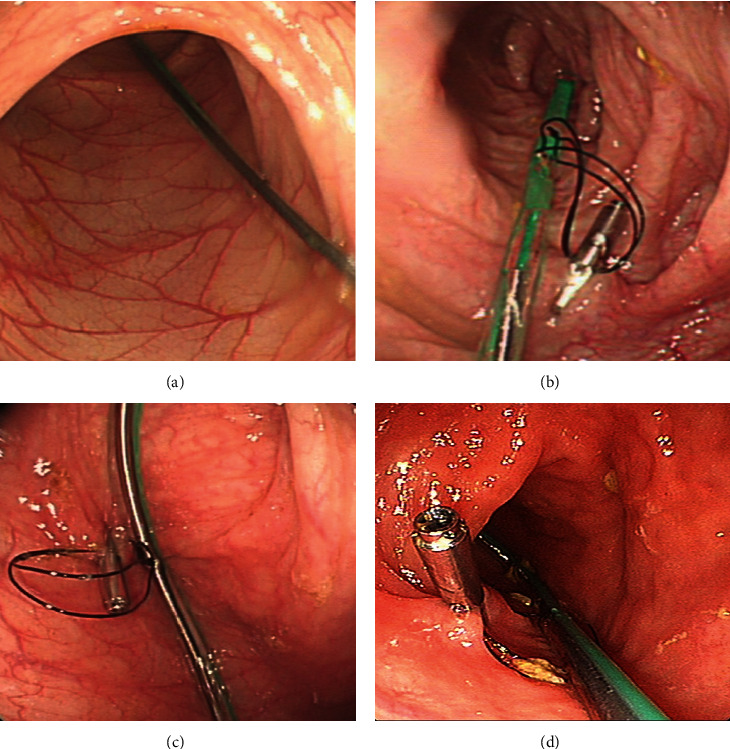
The colonoscope was reinserted to the target site using 3 endoscopic clips to fix the tube (a) on the upper intestinal wall at three sites. The “first site” (b) was located at the distal end of the tube, and the “second site” (c) and “third site” (d) were 10 cm apart.

Under intravenous anesthesia, routine colonoscopy was performed by using a colonoscope with a working channel ≥ 3.2 mm in diameter. After a complete assessment of the colon, a soft TET tube (2.7 mm OD, Figure 1(a)) was inserted into the colon through a paralubricated colonoscopy channel. Once the TET tube reached the target location (e.g., the cecum), the colonoscope was carefully withdrawn while maintaining the tube in place. Then, the colonoscope was reinserted to the target site using 1-4 endoscopic clips (ROOC-D-26-195-C, ≥10 mm) to fix the tube onto the colon wall using endoscopic clips at three sites. The “first site” (Figure 1(b)) was located at the distal end of the tube, and the “second site” (Figure 1(c)) and “third site” (Figure 1(d)) were 10 cm apart.

### 2.3. Healthy Donors and the Preparation of WMT

The WMT procedure was consistent with the Nanjing Consensus on the Methodology for Washed Microbiota Transplantation [24]. Healthy donors were aged between 18 and 25 years. First, healthy donors were asked to fill out a questionnaire that included medication history, family history, travel history, and residence history. Second, an interview was conducted to screen for mental health disorders such as anxiety and depression. Third, all healthy donors underwent examination of blood, urine, and feces, electrocardiogram, and X-ray.

The microbiota for WMT was extracted from the donated feces. Fecal samples were centrifuged and suspended three times using an intelligent microbial isolation system (GenFMTer; FMT Medical, Nanjing, China).

### 2.4. Protocol for WMT

After colonic TET intubation, each child received WMT for 6 consecutive days in a month (1 WMT course) for a total of 2 to 6 months, depending on their situation. Some children failed to receive WMT course for 6 consecutive months due to extraneous factors, such as financial constraints.

### 2.5. Clinical Evaluation of the Feasibility and Safety of Colonic TET

Relevant clinical data were retrospectively collected, including preprocedure bowel preparation methods, TET tube placement time, catheter placement success rate, and TET tube retention time (defined as the time from TET tube placement to natural detachment). Clinical data were collected, including ABC score (Aberrant Behavior Checklist score) and CARS score (Childhood Autism Rating Scale score). To satisfy the need for a full course of WMT treatment, TET should be kept for at least 6 days, and those less than 6 days are considered to be unmet clinical needs.

Parents of participants were retrospectively provided a questionnaire to assess their perceptions of colonic TET and the participants' responses to colonic TET placement. This data was used in combination with the description of participants' discomfort symptoms during the clinical course in the progress notes. Details of postintubation complications (such as fever, abdominal pain, abdominal distension, and diarrhea), status and changes in movements, and activities after intubation were collected comprehensively. Status during extubation (such as compliance, complications, and adverse events), participants' tolerance with TET, and overall parental satisfaction were also recorded. Participants' overall fitness level for TET was assessed based on the parental descriptions, and the psychiatric and gastrointestinal symptoms were recorded in progress notes. Data on the seniority level of the endoscopist (high-seniority endoscopist (>5 years of experience) versus low-seniority endoscopist (<5 years of experience)) and the time of TET catheterization were also collected.

### 2.6. Statistical Analysis

Data analyses were performed using SPSS software (version 22.0; SPSS Inc., Chicago, Illinois). Nonnormally distributed continuous variables were presented as median (interquartile range), and between-group differences were assessed using the nonparametric Wilcoxon rank sum test. Categorical variables were presented as frequency and percentages. *P* values < 0.05 were considered indicative of statistical significance.

## 3. Results

### 3.1. Participant Characteristics

A total of 38 participants (mean age: 6.3 years) were included in this retrospective study. A total of 124 TET intubation procedures were performed in this cohort: 25 procedures (20%) in 9 females and 99 procedures (80%) in 25 males (Table 1).

### 3.2. Feasibility of Colonic TET in Children with Autism

#### 3.2.1. Bowel Preparation Regimens

Bowel preparation regimens were oral laxative plus an enema. According to the child's acceptance of the bowel preparation regimen, oral laxatives included compound polyethylene glycol electrolyte, 20% mannitol, or lactulose.

#### 3.2.2. Feasibility of Colonic TET in Children with ASD

A total of 38 participants underwent 124 colonic TET catheterization procedures. In 123 procedures (99%), the TET tube allowed the completion of WMT treatment for 6 consecutive days. The average time of catheterization was 15 minutes, and the success rate was 100% (124/124). Accidental detachment of the tube within 6 days occurred only in 1 out of the tubes (0.8%). The average retention time of 124 procedures was 8.03 days. The tube was detached spontaneously after the end of the treatment course in 118 procedures (95.2%). No tube blockage was observed during the tube indwelling process (Table 2).

#### 3.2.3. Difference between High-Seniority Endoscopist and Low-Seniority Endoscopist in the Colonic TET Tube Indwelling Practice

There was no significant difference between high-seniority endoscopist and low-seniority endoscopist with respect to the tube placement time, success rate of tube insertion, and the proportion of patients whose TET was kept for 6 days or longer (*P* > 0.05).

### 3.3. Safety, Degree of Comfort of the Colonic TET, and Parental Satisfaction

In this study, only 1 (0.8%) patient developed transient low-grade fever (37.5°C) after intubation, and the temperature dropped to normal levels after drinking water and bathing. Four instances (3.2% procedures) of postprocedure abdominal pain were relieved without special treatment, and no serious complications (intestinal perforation, intestinal bleeding, sepsis, bacteremia, abdominal distension, or diarrhea) were observed. There was no obvious impact of TET on children's daily life, and there was no tube removal due to complications.

According to the responses to the questionnaire, the parents of the participants were very satisfied with 118 procedures (95.2%) and satisfied with 6 procedures (4.8%) (Table 3).

## 4. Discussion

An increasing body of evidence has demonstrated an important effect of intestinal dysbiosis on gastrointestinal symptoms and neurodevelopmental dysfunction in ASD patients. Studies have shown that FMT can improve gastrointestinal and ASD symptoms in ASD patients without causing serious complications [25]. However, FMT treatment often requires repeated transplantations. Therefore, development of a safe and effective transplantation method is a key imperative. Colonic TET was reported to be a novel, safe, and convenient method for repeated WMT in children. Due to their young age and the impact of the disease itself, children with ASD face challenges in daily life and generally have worse communication, coordination, and compliance than non-ASD children. In addition, children with ASD have a higher incidence of gastrointestinal symptoms, including constipation, diarrhea, abdominal pain, and bloody stools [26]. Thus, compared with non-ASD children, children with ASD undoubtedly experience more difficulties with TET intubation, both before and after intubation. To this end, this study retrospectively investigated 124 via-colonic TET catheterization procedures performed in 38 children with ASD. We observed a 100% success rate in this cohort. Moreover, 6 consecutive days of WMT treatment was successfully completed with 123 TET tubes (99%). After TET catheterization, the mean retention time of the TET tube was 8.03 days, and no instances of tube blockage occurred during the retention period. These findings suggest that TET catheterization for WMT is feasible in children with ASD.

The safety and comfort of TET catheterization is another key aspect. In this study, there were only 10 instances (8%) of mild anal discomfort, 4 instances (3.2%) of mild abdominal pain, and 1 instance of low-grade fever. There were no other serious adverse events such as intestinal perforation, bleeding, bacteremia, abdominal pain, abdominal distension, or diarrhea. There was a high level of parental satisfaction. The parents did not report any specific impact of tube placement on routine activities such as sitting, walking, defecation, or playing. This indicated that TET catheterization is safe and comfortable for children. The safety data of the TET tubes in this study are similar to those reported in a study on non-ASD children by Professor Zhang [17].

Physiologically, intestines in children are shorter, narrower, and more delicate than those in adults [27]. Therefore, endoscopists should be more careful while performing colonoscopy in children; this procedure requires a better colonoscopic TET catheterization technology and perhaps a longer operation time. In this study, there was no significant difference in operation time or success rate between physicians with different seniority levels. The average time required for the colonoscopic TET catheterization procedures was only 15 minutes. These findings suggest that colonoscopic TET catheterization in children with ASD is a safe and simple operation that low-seniority endoscopists can master after training.

However, the retrospective study design and the small sample size are key limitations of our study. Our results should be verified in a larger prospective study.

## 5. Conclusions

In general, for the repetitive administration of WMT in children with ASD, the colonic TET procedure achieved a 100% success rate. In 99% of the procedures, 6 consecutive days of WMT were successfully performed with TET tube placement. Moreover, the tubes did not cause any obvious discomfort to the child, and the incidence of spontaneous detachment was exceedingly rare. Further, the indwelling tube did not impede the daily activities of the child or induce any adverse events. These results demonstrate the safety and feasibility of colonic TET, a new interventional endoscopic technique for WMT, in children with ASD.

## Figures and Tables

**Table 1:** Characteristics of the study population.

Items		Result
Age (mean ± SD)		6.37 ± 3.41
BMI (kg/m^2^, median, IQR)		15.77 (14.57, 17.48)
Male (*n*, percent)		99 (80%)
Female (*n*, percent)		25 (20%)
ABC score		69.00 (50.00, 78.00)
CARS score		37.00 (34.00, 39.37)
WMT course (*n*, percent)	2 courses 3 courses 4 courses 5 courses 6 courses	2 (5.3%) 3 (7.9%) 1 (2.6%) 8 (21.1%) 24 (63.1%)

Data presented as mean ± SD, median (IQR), or *n* (%). BMI: body mass index; ABC: Aberrant Behavior Checklist; CARS: Childhood Autism Rating Scale.

**Table 2:** Feasibility of colon TET intubation in children with ASD.

Item	Result
Time required for insertion of tube (minutes; median, IQR)	15.15 (10.00, 17.00)
Success rate of tube insertion (*n*, percentage)	100%
Retention time of TET tubes (days; median, IQR)	8.03 (8.00, 9.00)
The tube detached spontaneously after the end of the treatment course (*n*, percent)	118(95.2%)
TET tubes were retained for at least 6 days (*n*, percent)	123 (99%)

Data presented as median (IQR) and *n* (%).

**Table 3:** Complications, impact of colonic TET catheterization on the daily life, and the parental satisfaction of children with ASD.

Item		Result
Complaints related to indwelling tube	Fever	1 (0.8%)
	Abdominal pain	4 (3.2%)
	Mild anal discomfort	10 (8%)
	Bloating or diarrhea Anal pain	0

Impact of TET on children's daily life	Sitting/walking, defecation, or playing	0

Parental satisfaction	Unsatisfactory	0
	Satisfactory	6 (4.8%)
	Very satisfactory	118 (95.2%)

## Data Availability

The data used to support the findings of this study are available on request from the corresponding authors. The data are not publicly available due to privacy or ethical restrictions.
